# 3D Printing Bioinspired Ceramic Composites

**DOI:** 10.1038/s41598-017-14236-9

**Published:** 2017-10-23

**Authors:** Ezra Feilden, Claudio Ferraro, Qinghua Zhang, Esther García-Tuñón, Eleonora D’Elia, Finn Giuliani, Luc Vandeperre, Eduardo Saiz

**Affiliations:** 1 Centre of Advanced Structural Ceramics, Department of Materials, Imperial College London, London, UK; 2 Hong Kong University of Science and Technology, HK, China; 3 Materials Innovation Factory & School of Engineering, University of Liverpool, Liverpool, UK; 4 Department of Mechanical Engineering, Imperial College London, London, UK

## Abstract

Natural structural materials like bone and shell have complex, hierarchical architectures designed to control crack propagation and fracture. In modern composites there is a critical trade-off between strength and toughness. Natural structures provide blueprints to overcome this, however this approach introduces another trade-off between fine structural manipulation and manufacturing complex shapes in practical sizes and times. Here we show that robocasting can be used to build ceramic-based composite parts with a range of geometries, possessing microstructures unattainable by other production technologies. This is achieved by manipulating the rheology of ceramic pastes and the shear forces they experience during printing. To demonstrate the versatility of the approach we have fabricated highly mineralized composites with microscopic Bouligand structures that guide crack propagation and twisting in three dimensions, which we have followed using an original *in-situ* crack opening technique. In this way we can retain strength while enhancing toughness by using strategies taken from crustacean shells.

## Introduction

In order to improve the mechanical properties of materials their structure and architecture need to be carefully controlled at a range of scales from nm to cm. Natural materials offer blueprints for the design of composites to achieve this objective^1–4^. In particular they offer hints about how to combine strength and toughness, a goal that has been difficult to reach with synthetic composites as they are limited to relatively simple microstructures^5^. In contrast, many natural materials exhibit a remarkable level of complexity, with intricate structures at a range of length scales. Some well-studied examples include mammalian cortical bone^6^, nacre^7^ and certain areas of crustacean exoskeletons^8^. These materials exhibit microstructural designs able to direct crack propagation in three dimensions and to control fracture, generating strengths and toughness that are well beyond that of their constituent materials.

In recent decades 3D printing/additive manufacturing (AM) has progressed enormously, revolutionising the fields of rapid prototyping and the production of complex geometries. In some cases the precision and fine detail that 3D printing affords has enabled the production of parts which would have been impossible using traditional manufacturing techniques. Its value is now recognised across a wide range of sectors from aerospace to biomedical engineering. Most work has focused on printing molten polymers^9^ and metals^10^, while ceramics and glasses have received less attention. This is in part due to the inherent difficulties in melting these materials which limit the applicable AM techniques to powder bed processes^11^, wet processes^12^ and fused deposition^13^. Robocasting is a promising AM process which emerged two decades ago^14^, and was initially used to produce ceramic woodpile scaffold structures^15^. Green ceramic parts are built by extruding a filament of paste/ink through a computer-controlled nozzle instructed by a CAD model, in a similar manner to fused deposition of ceramics^13^. Objects are built up layer-by-layer, and thus far a wide range of ceramic^16^, metallic^17^, polymeric^18^, graphene^19^, bioactive^20^, and ferroelectric^21^ pastes have all been developed and printed.

While the complexity of the structure of natural materials has been known for some time, the advent of AM could offer the level of microstructural control needed to produce artificial analogues, and there have been a number of recent attempts to achieve this^22–29^. Sub-μm printing has become an increasingly mature field^30^, and while such detailed additive processes have demonstrated fantastic levels of control, and the final parts can have remarkable properties, they are impractical for producing macroscopic objects due to extremely slow printing rates. Thus, a technique is needed which combines μm-scale structural control with practical, macroscopic printing speeds. Robocasting is a strong candidate to achieve this goal. The shear forces arising from extrusion have been shown to align short carbon fibres in an epoxy matrix during printing, giving close control over fibre orientation within the part^31^. Magnetic fields have also been used to precisely align functionalised platelets during robocasting for similar purposes^32^. When combined with other processes robocasting also facilitates the production of a large range of composite systems with controlled architecture at multiple length scales. For example, printed ceramic scaffolds with controlled porosity have been infiltrated with liquid metals^33^ and liquid polymers^34^ to create a new class of interpenetrating phase composites. However, several challenges still persist as there are restrictions on the degree of structural control or the part composition. In particular many of them are limited to composites with relatively low contents of the reinforcing ceramic phase. These materials have only just begun to probe the vast number of possibilities of tuned composite systems which 3D printing has made viable at relatively low cost.

Here we use robocasting to produce a range of complex ceramic/polymer composite systems based on aligned alumina platelets. One key feature unique to this work is that the printed components contain a high volume fraction of ceramic material, as is the case with many natural materials, a feat that has yet to be achieved with other techniques. We manipulate the rheology of the ceramic paste and the shear forces during printing to build macroscopic composites with highly textured microstructures on multiple length scales, inspired by a number of natural materials such as bone, nacre, and wood. As a proof of concept we show how these microstructures can be used to rationally control crack propagation and twisting in three dimensions. In this way it is possible to replicate natural strategies and build highly mineralized materials that retain strength while enhancing toughness. Our approach enables the fabrication of samples on scales which allow implementation of an original *in-situ* double cantilever mechanical test in order to follow the interaction of cracks with different microstructural features.

### Rheology and Platelet Alignment

**Figure 1:**
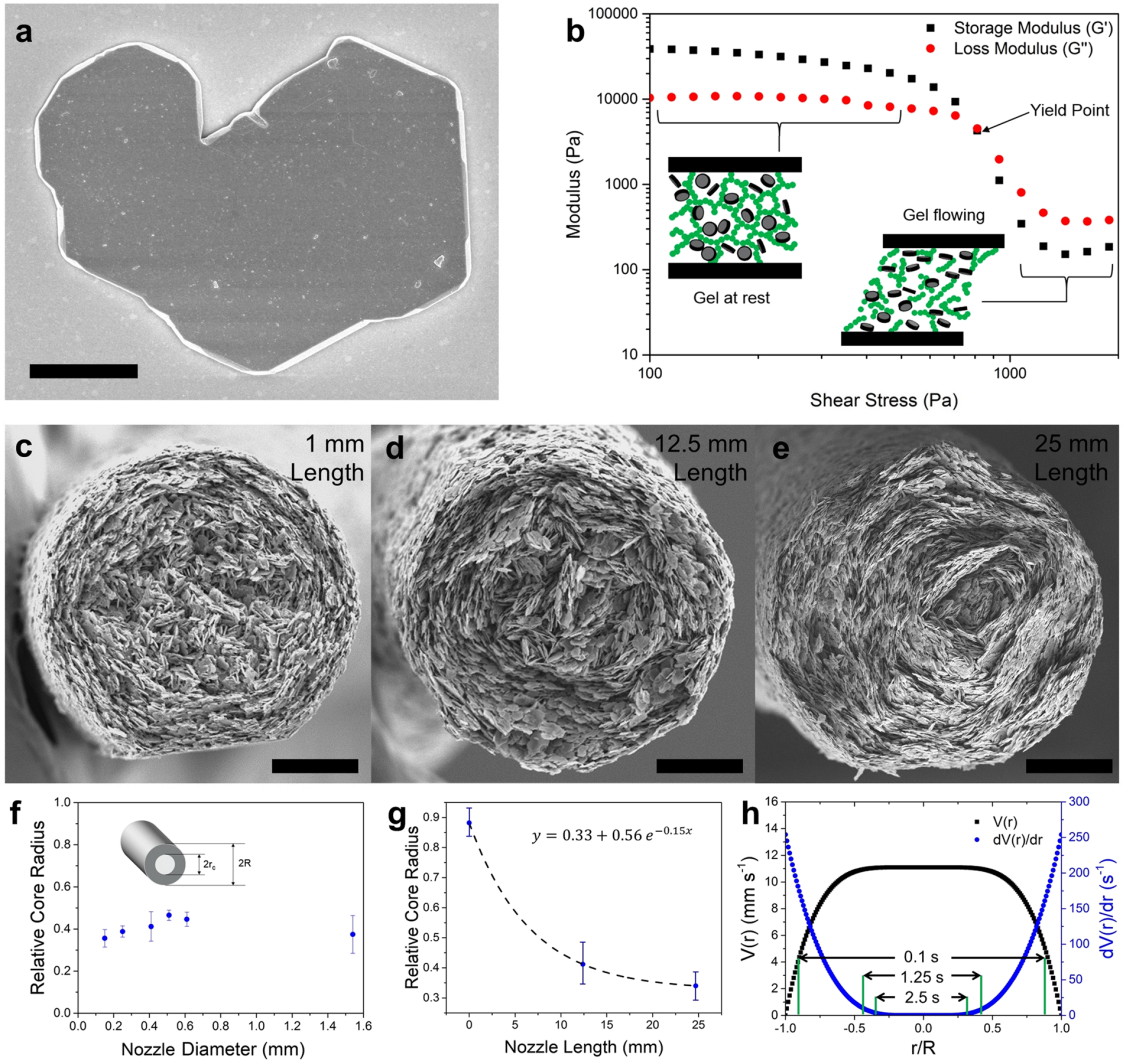
Rheology of platelet pastes and platelet alignment during extrusion. (**a**) SEM image of a single alumina platelet. Scale bar 2 μm. (**b**) Dynamic mechanical analysis of the platelet laden printing paste, showing the paste stiffness and yield stress. (**c**,**d**,**e**) SEM images of filaments printed using different nozzle lengths, showing the effect of nozzle length on the level of concentric platelet alignment. Scale bars 30 μm. (**f**) Measured relative core radius plotted against the nozzle radius, demonstrating invariance. (**g**) Measured core radius plotted against nozzle length, showing an exponential relationship. (**h**) Velocity profile, *V*(*r*), and velocity gradient, *dV*(*r*)*/d*(*r*) across a 0.41mm nozzle, computed using Equations 2 and 3. The experimentally measured core diameters are marked after the paste is subject to the velocity gradient for three amounts of time, corresponding to the three lengths of printing nozzles (1mm nozzle: 0.1 s, 12.5 mm nozzle: 1.25 s, 25 mm nozzle: 2.5 s).

The pastes used for 3D printing consist of microscopic hexagonal alumina platelets (~5 μm diameter, 0.5 μm thickness, see Fig. 1a) and submicron alumina powder at a 7:3 wt% ratio, mixed with a water-based hydrogel of Pluronic F-127 and 0.5 wt% dispersant to lower the viscosity. The pluronic-F127 hydrogel allows the paste to accept large fractions of alumina platelets, exceeding 30 vol%, resulting in a high volume fraction of ceramic material in the final printed parts. By tailoring the solids loading and hydrogel content, pastes based on the pluronic hydrogel have been shown to readily give the appropriate rheological properties for 3D printing^35^, which requires shear thinning behaviour, stiffness >10 KPa and a yield point >50 Pa. Figure 1b illustrates the shear thinning characteristic of the optimised paste and that the stiffness at rest amounts to 38 kPa with a yield point of 885 Pa. The latter compares favourably with the minimum rheological requirements for robocasting as empirically determined in previous work^35^.

The rheology of these pastes can be described by the Herschel-Bulkley model^36^;

1 $$\tau ={\tau }_{y}+K{\dot{\gamma }}^{n}$$

where *K* is the viscosity parameter, n is the shear thinning coefficient and $\dot{\gamma }$ is the shear rate. Pastes based on the Pluronic gel exhibit a yield stress (*τ*_*y*_ > 0) and shear thinning behaviour (0 < *n* < 1). The gel acts as a carrier for the ceramic powder and platelets, forming a soft network which breaks when sheared, allowing the paste to flow through a nozzle, but reforms when the stress is removed, allowing the printed part to maintain its shape. For the paste used here *K* is 780 Pa.s and *n* is equal to 0.3.

Upon extrusion through printing nozzles ranging in diameter from 100–510 μm, a distinct structure was observed in the printed material, shown in Fig. 1c–e. Nearly all of the platelets are aligned in the printing direction. In the outer region of the extruded filament, the platelets also align with the normal of the platelet face perpendicular to the nozzle wall, giving concentric circles of platelets^37^, while near the centre of the extruded filament the concentric alignment of platelets is more random. Within the range studied, the nozzle radius does not significantly alter the fraction of the filament where the platelets orient, Fig. 1f, while as the length of the nozzle increases, the radius of the randomly aligned core shrinks as illustrated in Fig. 1g.

The combination of the flow properties of the paste and the extrusion conditions determine the degree of platelet alignment. Approximating the printing nozzle as a uniform channel with a circular cross-section, the velocity, *V*, at a point across the radius $r$ can be calculated by applying the constitutive equation for Herschel-Bulkley fluids to the Navier–Stokes equations^38^:

2 $$V(r)=\frac{n\,R}{{\tau }_{w}(n+1)}{(\frac{1}{K})}^{\frac{1}{n}}[{({\tau }_{w}-{\tau }_{y})}^{\frac{n+1}{n}}-{(\frac{{\tau }_{w}r}{R}-{\tau }_{y})}^{\frac{n+1}{n}}]$$

where $R$ is the total radius of the channel and ${\tau }_{w}$ is the drag of the wall, calculated from the overall pressure drop across the length of the nozzle;

$${\rm{\Delta }}P=\frac{2{\tau }_{w}L}{R}$$

where *L* is the length of the nozzle. This gives a parabolic velocity profile (Fig. 1h) such that the velocity is at a minimum closest to the nozzle wall, and constant and maximum across a core region where the shear stress is lower than the paste’s yield stress. Upon differentiation the radial velocity gradient can be obtained;

3 $$\frac{dV(r)}{dr}=\,{(\frac{1}{K})}^{\frac{1}{n}}[{(\frac{{\tau }_{w}r}{R}-{\tau }_{y})}^{\frac{1}{n}}]$$

The velocity gradient is responsible for the concentric alignment: any platelets aligned approximately radially (the plate normal parallel to the nozzle wall) will experience a torque and rotate, whereas, tangentially aligned platelets do not straddle the velocity gradient, and therefore will not be affected by it. This results in tangentially oriented platelets dominating in number during extrusion. According to Equation 2 a core of material at the centre of the nozzle should show no alignment (where the velocity gradient is equal to zero, and the shear stress is below the yield stress) and the radius of the nozzle should not affect the extent of alignment. Furthermore, increasing the length of the nozzle should improve the degree of alignment as longer nozzles increase the amount of time that the paste is subjected to the velocity gradient. Therefore platelets which are positioned in areas which experience a low velocity gradient (such as those close to the centre of the nozzle) will have time to align, resulting in a smaller misaligned core. For example, when using the long nozzle the platelets experience the velocity gradient for 2.5 seconds. In the short nozzle the platelets experience the velocity gradient for just 0.1 seconds, so only the platelets in the highest velocity gradients (nearer to the nozzle wall) have enough time to align. Experimentally each of these predictions are validated. However, for smaller diameter nozzles (where the radius is <20 times the diameter of the platelets) the diameter of the core becomes proportionally smaller due to scaling effects with the nozzle wall becoming more significant.

### Ceramic-Polymer Composites

**Figure 2:**
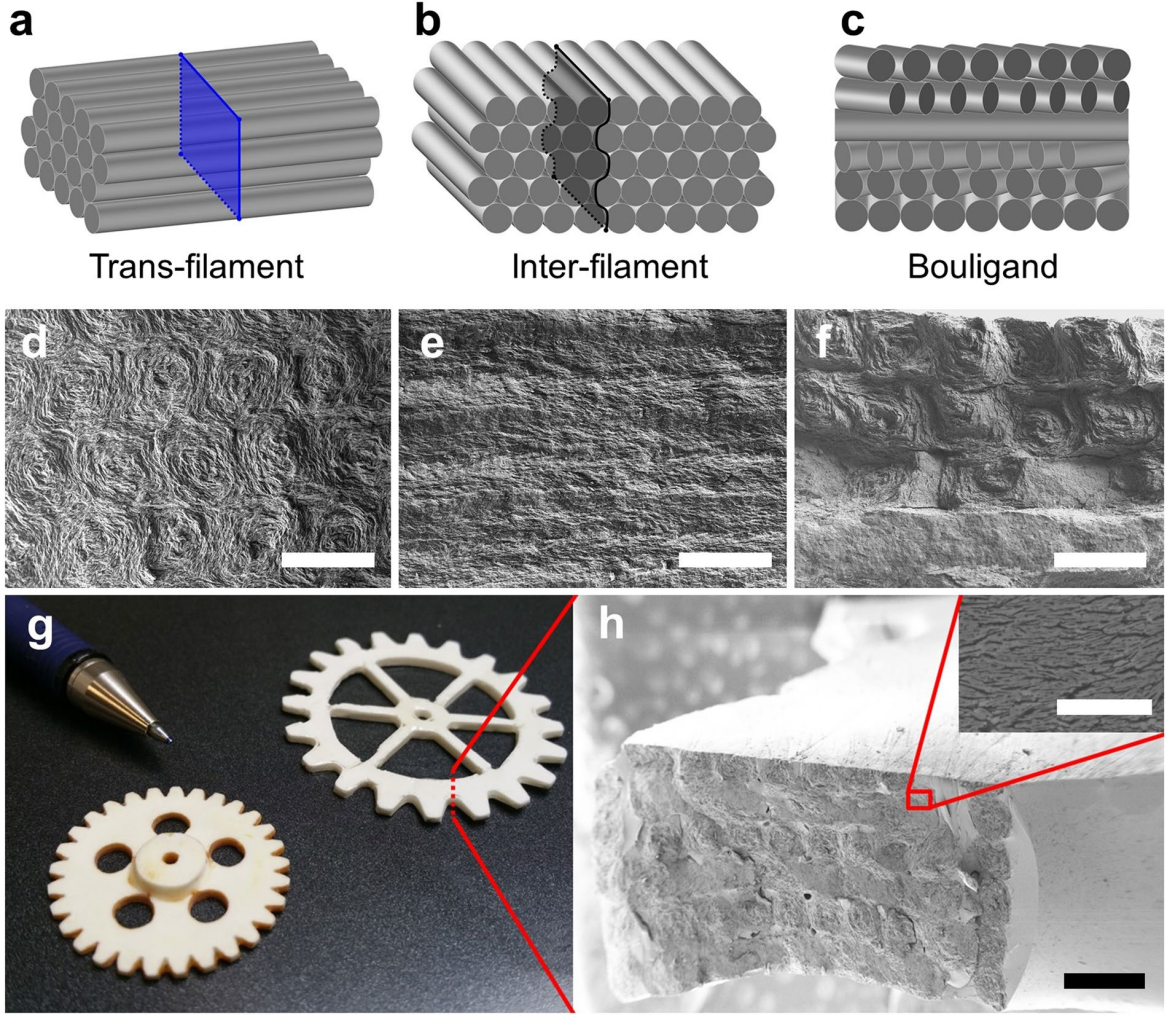
Microstructures of bioinspired platelet-epoxy composites produced via robocasting of platelet pastes and comparison to natural analogues. (**a**,**b**,**c**) Schematic diagrams of the three morphologies (trans-filament, inter-filament and Bouligand) produced by controlling the printing direction, with fracture direction indicated. (**d**,**e**,**f**) SEM images of fracture surfaces of the three composites, infiltrated with epoxy. Scale bars 400 μm. (**g**) Photograph of composite parts produced from arbitrary CAD files to demonstrate the flexibility of the technique. (**h**) SEM images showing the microstructure of the printed part. Scale bars 10 and 500 μm.

Following 3D printing and drying, isostatic pressing can be used to increase the volume fraction of platelets and powder in the green part, from ~50 vol% to ~64 vol%. All of the parts printed for mechanical characterisation were printed using the conditions which achieved maximum platelet alignment (the smallest disordered core). The addition of alumina powder to the printing paste allows the platelets to sinter, as the flat platelet faces will bond poorly on their own. Individual platelets have been shown to possess very high strength on the order of 5 GPa^39^, and after sintering the preforms are strong (~50 MPa) and easily handled, simplifying subsequent processing. The remaining ~36 vol% of porosity can be infiltrated with a second phase to build a composite. We filled the preforms with epoxy resin via vacuum infiltration, leading to composite parts with a final porosity below 0.5% as estimated from the Archimedes density. The printing process allowed a range of part geometries to be produced from CAD files, see Fig. 2h.

**Figure 3:**
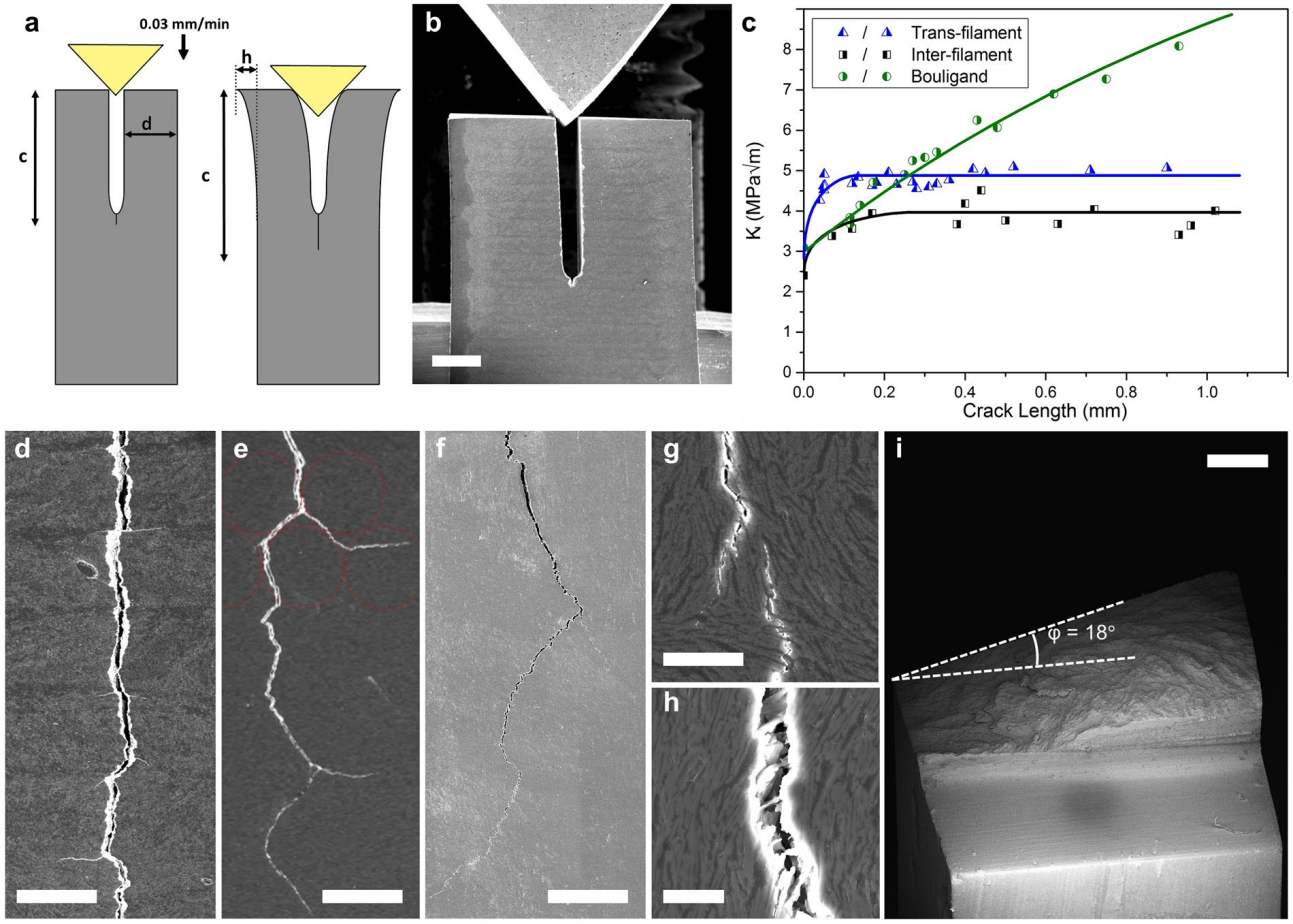
*In-situ* double cantilever beam testing of bioinspired platelet-epoxy composites. (**a**,**b**) Diagram and SEM image of the *in-situ* DCB setup. Scale bar 1mm. (**c**) Crack resistance curves of each composite, plotted from 6 tests. SEM images showing: (**d**) delamination of layers during trans-filament fracture, scale bar 300 μm, (**e**) deflection around filaments during inter-filament fracture, scale bar 300 μm, (**f**) crack deflection in the Bouligand structure, scale bar 300 μm, (**g**) large scale bridging, scale bar 20 μm, (**h**) small scale bridging of individual platelets, scale bar 10 μm, (**i**) SEM fractography of the Bouligand material showing a twist of the crack plane, scale bar 0.5 mm.

The orientation of the printed filaments (and therefore the orientation of the platelet alignment) as shown in Fig. 2d–f, has a large effect on mechanical properties such as bending strength and fracture toughness, as outlined in Table 1. Our approach allows us to quantify the degree of anisotropy in the materials by testing samples with crack propagation across the extruded filaments (trans-filament) or between the extruded filaments (inter-filament). Both strength and toughness were found to be higher in the trans-filament direction. Details of the toughening mechanisms acting in these composites were revealed by *in-situ* double cantilever beam testing inside an SEM (see supplementary information for videos). This test allows long (>1 mm) crack paths to be closely monitored in high resolution and correlated to the stress intensity, *K*_*I*_, to obtain crack resistance curves (R-curves) for each printing direction, shown in Fig. 3. In this way it is possible to evaluate the effect of large mesostructural features on crack propagation, compared to traditional bending tests with relatively thin beams which have limited applicability in accurately measuring *K*_*I*_ in such long cracks. The R-curve varies significantly between the different printed structures (Fig. 3c). For the trans- and inter-filament directions, *K*_*I*_ rises very steeply before levelling off after ~50 and ~200 μm respectively. In the trans-filament material the rise is primarily due to bridging, shown in Fig. 3g,h. Evidence for bridging at short crack lengths is shown in the supplementary information. As the crack grows, platelet bridges behind the crack tip are pulled out and so the *K*_*I*_ saturates; 50 μm is roughly the length over which pull out is observed. Meanwhile crack deflection is likely to be the main contributor to toughness in the inter-filament sample, see Fig. 3e. The crack should reach maximum deflection after deflecting around one filament. This is consistent with the fact that the R-curve for this material saturates after ~200 μm and the diameter of each filament is ~300 μm.

**Table 1:** Bulk mechanical properties of each composite material.

Orientation	Flexural Strength (MPa)	*K*_*IC*_ (MPa√m)	Young’s Modulus (GPa)	Hardness (GPa) (perpendicular/parallel to filaments)	Compressive Strength (MPa)	Density (gcm^−3^)
Trans-filament	202 ± 10	3.0 ± 0.3	99.1 ± 0.6	3.04 ± 0.70/2.52 ± 0.12	452	2.86
Inter-filament	125 ± 12	2.4 ± 0.6				
Bouligand	159 ± 42	3.1 ± 0.6		—	435

The composites consist of a strong network of alumina platelets sintered together at their points of contact by the alumina powder. This network imparts a high young’s modulus, as seen in Table 1, in the same range as many engineering alloys. Another consequence of this network is reasonably high hardness. Like strength and toughness, the hardness of our composites is anisotropic, with the direction perpendicular to the printing direction exhibiting ~20% higher hardness. This is because when indenting parallel to the printing direction, the platelets are generally oriented with the platelet-matrix interface in the loading direction. This allows the material to fail via buckling/sliding of the interface, lowering the hardness.

### Mesostructural Control- Bouligand Composites

The flexibility of the robocasting process allows for a high level of crack propagation control. Printed bars where the raster direction of each layer is rotated 30° with respect to the previous layer were produced, resulting in a highly mineralized Bouligand-type microstructure (Fig. 2c and f). These type of structures are often found in natural materials, such as the dactyl club of the mantis shrimp, due to their ability to enhance impact resistance and toughness^8,40^. The primary mechanisms responsible for these improvements are thought to be the smooth transition of elastic modulus between rotated layers as well as cracks propagating helically. This greatly increases the surface area of cracked material and changes to a less favourable mode of crack propagation, increasing the energy absorbed during fracture^8^. Synthetically, crack bending has been effectively used to control fracture in two dimensions^41^. However, it has been very difficult to replicate this degree of rotational control in synthetic three dimensional ceramic-based composites. In ceramic composites weak interfaces are often used to deflect cracks, but these are usually randomly distributed in the microstructure, with the exception of laminates which can only provide deflection in one particular direction (where the weak path is placed 90° with respect to the crack propagation)^1,42,43^. Printing allows us to rationally direct fracture by playing with the relative orientation of the printed filaments. As the inter-filament direction is less strong and less tough than the trans-filament direction, cracks will preferentially grow on this plane, resulting in the “delamination” shown in Fig. 3d. This means that the crack can be directed and controlled, twisting and tilting onto planes which are more difficult to grow on. The relationship between the strain energy release rate that the crack tip experiences (*G*_*I*_) and the angle of this twist ($\phi$) or tilt (*θ*) is given by^44^:

4 $${G}_{I}(\phi )=\,{G}_{IC}\,co{s}^{2}(\phi )$$

5 $${G}_{I}(\theta )=\,{G}_{IC}\,co{s}^{2}(\frac{\theta }{2})$$

As the crack grows, it encounters the boundaries between printed layers rotated 30° with respect to one another. Whether the crack either twists, or continues on its current plane is determined by the relative magnitude of the strain energy release rate and the fracture resistance along the different paths. The *G*_*IC*_ of the material (calculated from the $\,{K}_{IC}$) is fairly anisotropic, with the inter-filament critical strain energy release rate,$\,{{G}_{IC}}^{inter}$, only 64% of the trans-filament critical strain energy release rate, ${{G}_{IC}}^{trans}$:

6 $${{G}_{IC}}^{inter}/{{G}_{IC}}^{trans}\approx 0.64$$

Assuming an isotropic elastic modulus, a crack propagating in the trans-filament direction will twist onto a path of inter-filament propagation if the decrease in crack resistance given by equation 6 is larger than decrease in crack driving force from equation 4. Thus in theory it is possible to guide the crack, at the 100 s of μm scale, using only the material’s microstructure, to a twist angle of $co{s}^{-1}\sqrt{0.64}$ = 36.9°. This has the effect of raising the R-curve by a factor of 3 with respect to the other inter- and trans-filament materials, as seen in Fig. 3c. In a similar vein, the crack may potentially tilt up to $2\,\ast \,co{s}^{-1}\sqrt{0.64}$ = 73.7° to maximise *G*_*I*_. This partially explains the behaviour illustrated in Fig. 3e, where the crack was observed to follow the perimeter of the printed filaments, often deflecting up to *θ* = 75°, and also the delamination behaviour, where the crack temporarily deflects up to *θ* = 90°, changing to entirely mode II fracture until the crack re-nucleates and returns to mode I in the next layer. SEM fractography, shown in Fig. 3i, revealed that the crack to only twists up to an angle of *φ*≈18°, but this angle may be increased by increasing the number of layers the crack travels through. This mechanism alone does not explain all of the increase in *K*_*I*_ for the Bouligand material presented in Fig. 3c compared to the other two structures, as a twist of $\phi$ = 18° accounts for only a 10% decrease in crack driving force. However, twisting will also generate friction between the crack surfaces as they open. As the crack grows, the area generating this friction should increase linearly, thus crack resistance should also increase linearly. It can be observed that, in opposition to the inter- or trans-filament fractures, the R-curve does not reach a plateau but rather rises steadily for distances up to 1 mm, which points to the triggering of a different toughening mechanism.

With a high volume fraction of the aligned reinforcing phase, these composites can exhibit higher strength and stiffness than traditional short carbon fibre reinforced epoxy composites (CFRP)^45^, while maintaining ease of manufacture. Due to the strong platelet network our composites have a much higher hardness and bulk modulus than CFRP^46^, and are expected to have improved wear resistance. They also exhibit comparable compressive strength to even long fibre CFRPs^47,48^. Meanwhile, the composites outperform the strength of natural analogues utilising similar toughening mechanisms (such as nacre and cortical bone) by a factor of two^2^. This is likely due to the strong bonding between platelets in our material, as well as the high strength of the polymer phase compared to natural polymers. The *K*_*IC*_ of these three materials are roughly comparable. The R-curve of bone is generally less steep, reaching lower *K*_*I*_ within the crack lengths studied here, while the R-curve of nacre is similar to our composites^2^.

In summary, we have used robocasting to build ceramic scaffolds with complex shapes and unique hierarchical structures in practical times and sizes. This is done by manipulating the velocity gradients during extrusion and the rheology of the pastes containing high loadings of anisotropic particles. Composites with high ceramic content were fabricated by infiltrating these preforms with a soft phase. The architecture of the printed filaments can be used to direct crack propagation (twisting and tilting) at the microscopic level in three dimensions with a degree of control that is not presently possible to achieve using other approaches. As an example we have shown how toughness can be increased by altering the fracture behaviour on a mechanistic level in microscopic ceramic Bouligand structures. In this way it is possible to tailor and improve the anisotropic properties and fracture resistance and reach mechanical properties which compare favourably with many engineering materials. These results show how the combination of additive manufacturing with microstructural control opens new possibilities in the control of fracture, enhancing toughness and defect tolerance while maintaining a high specific strength. This approach can offer new opportunities in the design and fabrication of structural, light-weight composites for diverse applications from aerospace to automotive. Further work will focus on decreasing the characteristic microstructural dimensions, implementing further toughening strategies (for example through the use of other particles such as fibres), as well as exploring other material combinations.

## Experimental Methods

Alumina powder (SMA6, Baikowski, FR) with an average diameter of 0.35 μm was sieved through 100 μm to break down large agglomerates. The sieved powder was mixed with alumina platelets with a thickness of ~0.5 μm and a diameter of ~10 μm provided by Advanced Nano Technologies Ltd., Australia, at a mass ratio of 3:7. 20 g of this powder mixture was then added to a Teflon flask with 8 g deionised water and 0.1 g of the dispersant Dolapix CA (Zschimmer & Schwarz, Germany). This was mixed by hand followed by sonication with a SciMED 200 W horn sonicator for 60 seconds to break agglomerates. 2.75 g Pluronic copolymer (F-127, BASF, Germany) was then added to make this slurry printable, followed by four rounds of mixing in a Thinky ARE-250 planetary mixer at 2000 revolutions per minute and 800 rotations per minute, for 2 minutes, allowing the container to cool between steps. Finally the paste is defoamed in the planetary mixer at 2200 revolutions per minute and 60 rotations per minute for 10 minutes. For larger masses of paste (>30 g) longer defoaming was required. This step is critical to remove bubbles from the paste which are often pervasive in pastes involving platelets. Pastes were then cooled again and transferred to 3 cm3 syringe barrels ready for printing. Overall the pastes are 65 wt% (31 vol%) ceramic material.

Rheological measurements were carried out on a TA Instruments Discovery HR-1 rheometer with a 40 mm parallel plate geometry, a 1 mm gap and a solvent trap to prevent drying. Flow ramps were conducted at strain rates of 0.02–200 s^−1^. Dynamic mechanical analysis (DMA) was carried out at 1Hz, varying the oscillation stress from 0.1–3000 Pa. The phase lag (*δ*) between peak shear stress and peak shear strain was determined automatically by the equipment’s software averaging over 10 oscillations. Yield stresses were calculated by observing the stress at which the storage modulus (*G’*) is equal to the loss modulus (*G”*) during DMA. The pressure drop in the nozzle was measured by using a Zwick iLine mechanical testing rig to apply a displacement rate to extrude the filament at 10 mm/s, and measuring the equilibrium force.

Aerobasic G-code was used to design the tool path to create the raster patterns of each layer. The G-code was visualised with RoboCAD (3dInks, USA). Printing was carried out on a robocaster system (3dInks, USA) onto PTFE substrates. A 40 mm dummy line (known as the “lead-in”) was printed immediately before the start of each part to ensure flow is homogenous as the part is printed. 40 × 4 × 3 mm^3^ test bars were printed in each orientation, using a 0.41 mm diameter, 25 mm length, steel nozzle. The print speed was 10 mm/s and the raster spacing was set to 0.34 mm which were determined to be appropriate printing parameters to ensure there are no defects between printed lines in previous work^35^. The temperature and humidity of the whole system was controlled by a custom built enclosure and a convection heater set to 23 °C while the measured humidity varied from 70–95%.

Printed parts were dried over 24 hours in a humidified enclosure to avoid cracking, followed by another drying step in a convection oven at 37 °C. Parts were then pressed at 300 MPa for 1 minute in an evacuated pouch using a Stanstead Fluid Power isostatic press. The pluronic was burnt out for 1 hour at 600 °C in air and sintering took place without pressure at 1550 °C for one hour in air. All heating and cooling rates were 300 °C h^−1^. Infiltration of Araldite resin (LY556, Huntsman llc, USA) into these preforms was achieved by thoroughly mixing the resin with XB 3473 hardener (Huntsman llc, USA) at a ratio of 100:23 by weight, as suggested by the manufacturer, and then pouring this mixture over the preforms and holding for >12 hours under vacuum. Curing took place at 180 °C for 1 hour in a convection oven followed by post-curing at 200 °C for 2 hours.

Samples were prepared for strength testing by polishing the composite bars down to 1 μm. For toughness measurements, a notch was made using a 0.84 mm diamond wafering blade followed by sharpening with a razor blade and 1 μm diamond suspension. The radius of curvature of the sharpened notch was generally 5–20 μm, however smaller surface defects at the bottom of the notch are likely to have smaller radii of curvature. Nonetheless, the bluntness of the notch potentially inflates the measured *K*_*IC*_ of these samples. Other methods to obtain a sharp notch, such as precracking, were not used as they would result in overestimating the *K*_*IC*_ due to toughening mechanisms being activated in the precracked material. A 4-point bend rig on a Zwick iLine universal testing machine was used with a displacement rate of 0.2 mm min^−1^ for the flexural strength measurements and 0.01 mm min^-1^ for single edge notched beam tests. 3 to 5 samples were tested for each data point. The same instrument was used with flat plates to test compression strength on 5 mm × 5 mm × 5 mm cubes at a rate of 1.3 mm min^-1^. R-curve measurements were conducted on samples machined into double cantilever geometries, see Fig. 3a,b. The total length, width and height of the samples was ~10 × 4 × 3 mm. The length of the arms was ~3.5 mm and a wide groove was machined along the back of the sample ~1 mm deep in order to prevent the crack from deviating towards the edge of the sample. A 2 kN Gatan *in-situ* mechanical testing stage was used with a custom machined steel wedge in an FEI Quanta FEGSEM. These test were carried out under displacement control with a displacement rate of the wedge of ~30 µm min^-1^. The displacement of the arms of the specimen was tracked separately to the crack tip for precision. In this setup, ${K}_{I}=\sqrt{\frac{3{E}^{2}{h}^{2}{d}^{3}}{4{c}^{4}}}$ where *E* is the Young’s modulus, *h* is the horizontal displacement of each arm, *d* is the half width of the sample and *c* is the length of the crack and arms^49^. As *K*_*I*_ is inversely proportional to *c* ^4^, the crack exhibits a high degree of stability, and can be grown over a long distance (>5 mm) in a very controlled manner. Hardness was measured using an Indentec instrument at 9.8 N load, using a near instantaneous loading rate, and a hold time of 10 seconds. The hardness was then determined by measuring the area of >10 indents in an optical microscope. Elastic moduli were measured using the impulse excitation technique with an RFDA instrument produced by IMCE, Belgium, and the values obtained by this method were used in all equations where *E* was needed.

Microstructures and fracture surfaces were examined by coating with gold and imaging with secondary electrons in a JEOL® JSM-6010 SEM at 20 kV. X-ray tomography was performed at the Diamond Light Source facility. All densities were measured by the Archimedes method.

## Electronic supplementary material

Supplementary Information

Figure 5s. SEM video showing an example of in-situ DCB testing of a trans-filament sample.

Figure 6s. SEM video showing crack propagation and deflection during in-situ DCB testing of a trans-filament sample.

Figure 7s. SEM video showing crack propagation and deflection during in-situ DCB testing of a trans-filament sample.

Figure 8s. SEM video showing crack bridging during in-situ DCB testing of an inter-filament sample.

Figure 9s. SEM video showing crack bridging and pull-out during in-situ DCB testing of a trans-filament sample.
