# Supplementary material for: 3D Printing Bioinspired Ceramic Composites
Source: Sci Rep. 2017 Oct 23;7:13759. doi: 10.1038/s41598-017-14236-9 (PMC5653810; doi:10.1038/s41598-017-14236-9)
Supplement: Supplementary file 1 — Supplementary Information [file 41598_2017_14236_MOESM1_ESM.pdf]

Supplementary Information

3D Printing Bioinspired Ceramic Composites

Ezra Feilden, Claudio Ferraro, Qinghua Zhang, Esther García-Tuñón, Eleonora D'Elia, Finn Giuliani, Luc Vandeperre, Eduardo Saiz

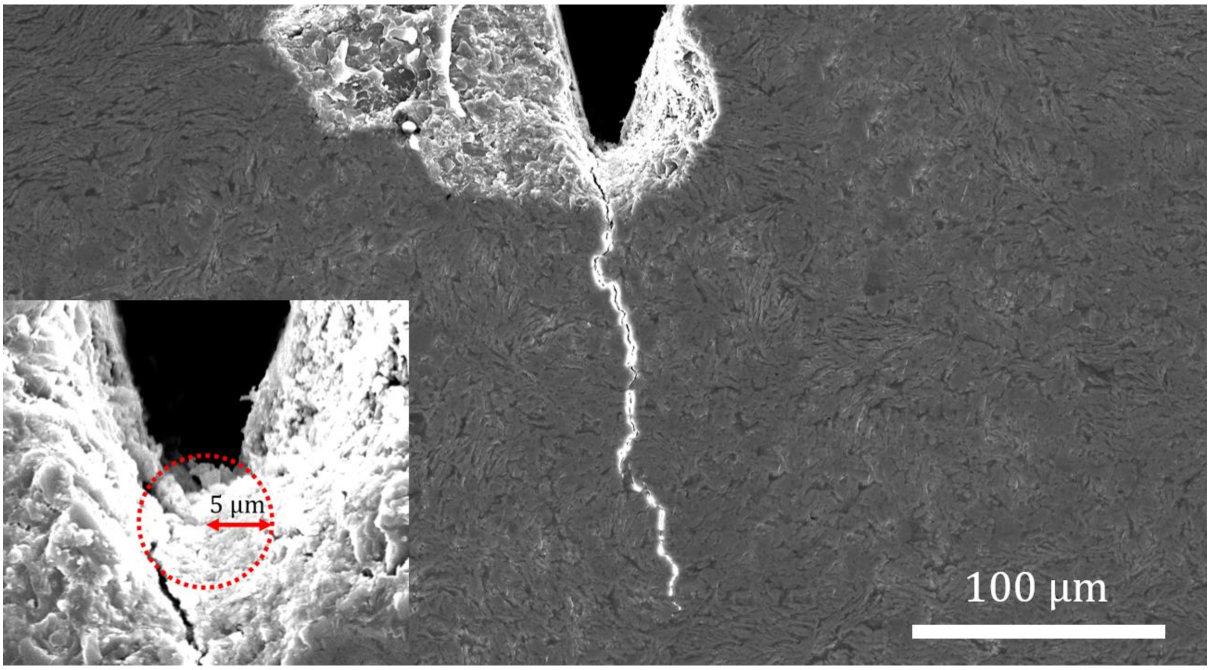

Figure 1s. SEM images showing a typical razor sharpened notch in a trans-filament sample. The radius of curvature of this notch is ~5 µm.

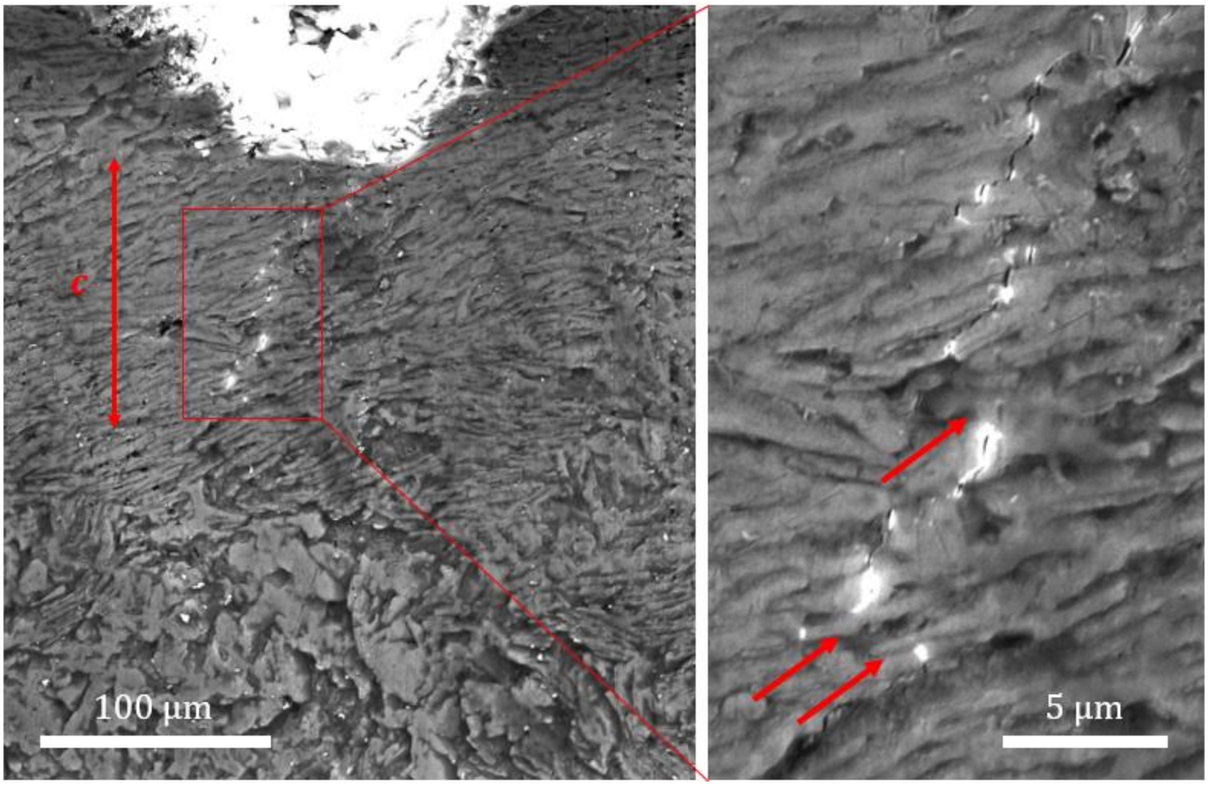

Figure 2s. SEM images of a crack in a trans-filament material, just after initiation, showing bridging material.

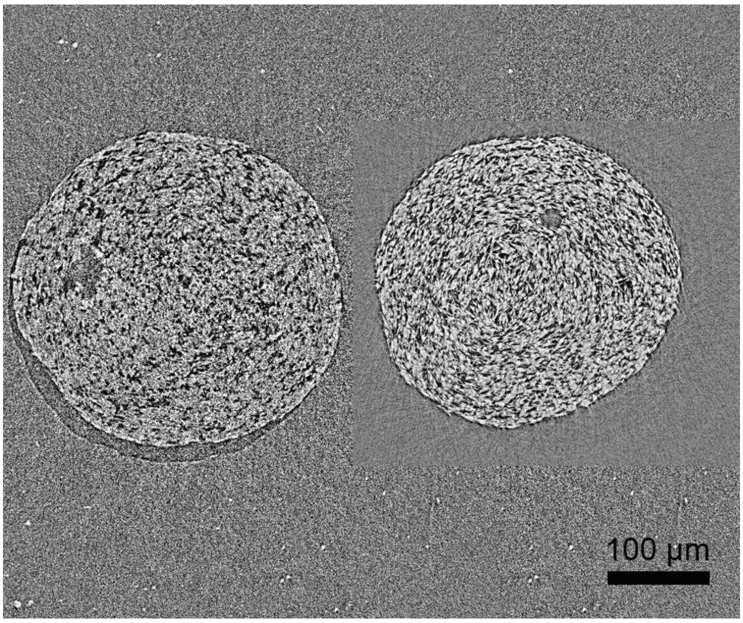

Figure 3s. X-ray tomography of a section of paste at the top of the long nozzle (left) and the bottom of the nozzle (right). An improvement in order can be somewhat observed.

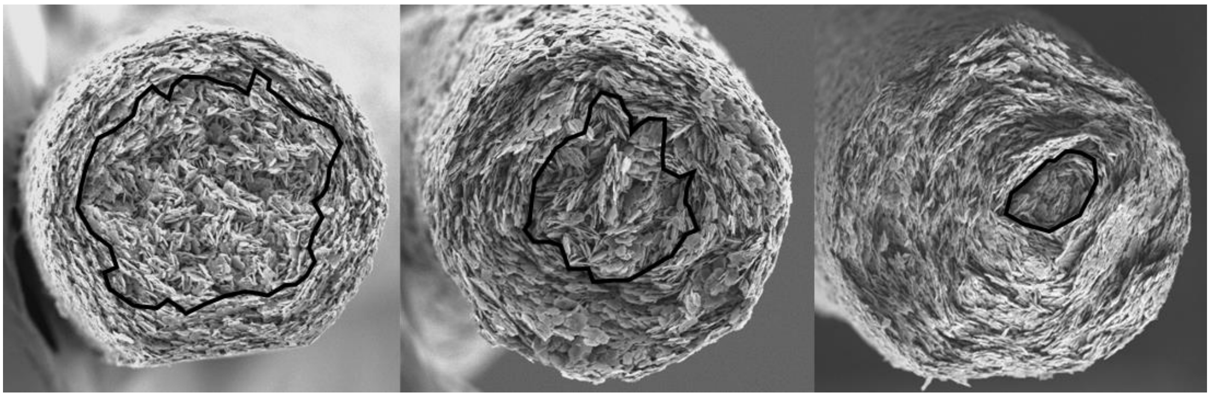

Figure 4s. Example measurement of the boundary of the unaligned core size when using three different nozzle lengths.

Figure 5s. SEM video showing an example of in-situ DCB testing of a trans-filament sample.

Figure 6s. SEM video showing crack propagation and deflection during in-situ DCB testing of a trans-filament sample.

Figure 7s. SEM video showing crack propagation and deflection during in-situ DCB testing of a trans-filament sample.

Figure 8s. SEM video showing crack bridging during in-situ DCB testing of an inter-filament sample.

Figure 9s. SEM video showing crack bridging and pull-out during in-situ DCB testing of a trans-filament sample.
